# Advances in Astrocyte Computational Models: From Metabolic Reconstructions to Multi-omic Approaches

**DOI:** 10.3389/fninf.2020.00035

**Published:** 2020-08-07

**Authors:** Janneth González, Andrés Pinzón, Andrea Angarita-Rodríguez, Andrés Felipe Aristizabal, George E. Barreto, Cynthia Martín-Jiménez

**Affiliations:** ^1^Departamento de Nutrición y Bioquímica, Facultad de Ciencias, Pontificia Universidad Javeriana, Bogotá, Colombia; ^2^Laboratorio de Bioinformática y Biología de Sistemas, Universidad Nacional de Colombia Bogotá, Bogotá, Colombia; ^3^Department of Biological Sciences, University of Limerick, Limerick, Ireland; ^4^Health Research Institute, University of Limerick, Limerick, Ireland

**Keywords:** computational model, astrocytes, system biology, data integration, high-throughput data, omics

## Abstract

The growing importance of astrocytes in the field of neuroscience has led to a greater number of computational models devoted to the study of astrocytic functions and their metabolic interactions with neurons. The modeling of these interactions demands a combined understanding of brain physiology and the development of computational frameworks based on genomic-scale reconstructions, system biology, and dynamic models. These computational approaches have helped to highlight the neuroprotective mechanisms triggered by astrocytes and other glial cells, both under normal conditions and during neurodegenerative processes. In the present review, we evaluate some of the most relevant models of astrocyte metabolism, including genome-scale reconstructions and astrocyte-neuron interactions developed in the last few years. Additionally, we discuss novel strategies from the multi-omics perspective and computational models of other glial cell types that will increase our knowledge in brain metabolism and its association with neurodegenerative diseases.

## Introduction

Astrocytes have gained a broad interest in neuroscience as they are essential for the maintenance of brain homeostasis and neuronal protection. Both in physiological conditions and disease, astrocytes are important in synaptogenesis, neurotransmitter release, cognition, neuroinflammation, glycogen storage, blood-brain barrier (BBB) formation, the clearance of toxic substances such as glutamate excess and K^+^ spatial buffering, and the release of trophic growth factors for neurons and other brain cells (Volterra and Meldolesi, 2005; Hamby and Sofroniew, 2010; Kimelberg and Nedergaard, 2010; Barreto et al., 2011; Parpura et al., 2011; Cabezas et al., 2012, 2014; Posada-Duque et al., 2014; Robertson, 2018). Moreover, astrocytic functions are based in an intimate relationship with neurons at both molecular and morphological levels through their endfeet, forming the tripartite synapse with presynaptic and postsynaptic neurons (Perea et al., 2009; Pérez-Alvarez and Araque, 2013; Coulter and Steinhäuser, 2015). In this aspect, astrocytic metabolic deregulation is a hallmark of neurodegenerative diseases and damaging processes such as amyotrophic lateral sclerosis (ALS), Alzheimer’s disease (AD), Huntington’s disease (HD), Parkinson’s disease (PD) and traumatic brain injury (Volterra and Meldolesi, 2005; Maragakis and Rothstein, 2006; Hamby and Sofroniew, 2010; Kimelberg and Nedergaard, 2010; Parpura et al., 2011).

The metabolic pathways modulated by astrocytes have been studied both experimentally and through the use of different computational models (Yu et al., 2002; Jin and Brennan, 2008; Sertbaş et al., 2014; Martín-Jiménez et al., 2017). However, there is still a lack of understanding of the metabolic relationship between these interactions and drug response, environmental agents, or pathological conditions. Therefore, the development of a comprehensive view of the astrocytic mechanisms involved in the brain-behavior requires a systemic approach, that can be assessed utilizing computational modelings, such as genome-scale metabolic models (GSMMs), which are created through an iterative process, integrating experimental evidence and computational approaches (Liu and Chen, 2010).

Computational models have been used successfully for testing an experimental hypothesis or guide wet-bench research, generating novel insights on the multiple mechanisms of the human brain (Lewis et al., 2010; Hyduke et al., 2013). Additionally, during the last 15 years, the adoption of system biology approaches, and the generation of genome-scale reconstructions of different cell types has played a critical role in the understanding of the complex mechanisms which couple specific metabolic profiles with cellular functions (Schuster et al., 2000; Agutter, 2005; Dada and Mendes, 2011; Edwards et al., 2011; Thiele et al., 2013).

Numerous reviews have focused on computational models as regards to calcium signaling in astrocytes and its modulation in synaptic transmission, gliotransmitter release and related processes (Volman et al., 2012; Linne and Jalonen, 2014; Oschmann et al., 2016; Manninen et al., 2018). In the present review, we focus on the current progress in computational models of astrocytic metabolism including genome-scale reconstructions, dynamical models, and multi-omic perspectives.

## Astrocytes Are Important for Brain Homeostasis

### Astrocyte Morphology and Reactive Gliosis

Astrocytes are the most versatile cells in the central nervous system (CNS) of vertebrates and constitute the glial cells, along with oligodendrocytes and microglia with the counting of 20–40%, 40–60%, and 10% or less respectively (Herculano-Houzel and Dos Santos, 2018; Verkhratsky and Butt, 2018; Verkhratsky and Nedergaard, 2018). Although classical studies categorized astrocytes into two groups, astrocytes are a heterogeneous group of star-shaped cells, with 11 main subtypes in mammalians. The first classical group contains the protoplastic astrocytes in the gray matter and numerous branches, and the second group is the fibrous astrocytes in the white matter, these are associated with myelinated axonal tracts and are related to the nodes of Ranvier. Moreover, recent studies classified astrocytes as surface-associated astrocytes, velate astrocytes, pituicytes, Gomori astrocytes, perivascular and marginal astrocytes, radial astrocytes, interlaminar astrocytes, polarized astrocytes, and varicose projection astrocytes, these last three cells subtypes are only in the brains of humans (Verkhratsky and Nedergaard, 2018; Souza et al., 2019). However, additional classes of astrocyte populations have been recognized based on glial fibrillary acidic protein (GFAP) and S100B labeling, including Muller glia, Bergmann glia, perivascular glia, ependymal glia and marginal glia (Matyash and Kettenmann, 2010).

Architecturally, astrocytes are coupled forming a similar tile-like organization associated with functional and morphological heterogeneity and have the ability to coordinate with the neighbor cells in the CNS. Moreover, they represent at least half of the synaptic contacts that are covered with glial processes in the human brain, and there these cells can exert contact with over 2 million tripartite synapses through their endfeet (Allen and Eroglu, 2017; Verkhratsky and Nedergaard, 2018). Furthermore, this architecture is pivotal for the astrocytic function in the metabolic regulation of the brain and the neurovascular coupling (Robertson, 2013). Interestingly, another role for astroglia is mounting brain defense against all types of pathological insults. In the last years, researchers focused on the role of reactive gliosis that involve complex reactions among cells of numerous linages as microglia (Burda and Sofroiew, 2014). However, there are other mechanisms modulated by astrocytes to protect the CNS under basal conditions and after injury. (a) Mediation of mitochondrial repair mechanisms by mitophagy of damaged mitochondria from neurons and return of healthy mitochondria to neurons. (b) Protection against glutamate toxicity across uptake of extracellular glutamate by amino acid transporter 2 (EAAT2) and glutamate transporter 1 (GLT-1). (c) Protection against redox stress through the activation of NrF2 and regulation of antioxidant genes. (d) Protection against glucose-induced metabolic stress by uptake of extracellular glucose for storage as glycogen. (e) Protection against iron toxicity by sequester free iron for storage with ferritin. (f) Maintenance of tissue homeostasis from DNA damage, through different DNA repair pathways like homologous recombination and non-homologous end-joining. (g) Modulation of the immune response by inhibition of T cells and monocytes (Bylicky et al., 2018). It is important to note that gliosis plays a role in responding to brain insults, but their inflammatory response and their activity in the regulation of immune cells is controversial (Lapuente-Chala and Céspedes-Rubio, 2018). Astrogliosis is a defensive reaction that involves both morphological and molecular changes as a response to injury, where astrocytes increase at the lesion site with an altered morphology (Kuroiwa et al., 2016). The proliferation of adjacent astrocytes implies an increased expression of the intermediate glial filaments such as vimentin, GFAP, nestin, and chondroitin sulfate proteoglycans (Hamby and Sofroniew, 2010; Bylicky et al., 2018). Different studies have shown that reactive gliosis is an important process for CNS during injuries and diseases as it helps in BBB remodeling, the release of vasoconstrictors and glutathione, and formation of a glial scar that inhibits axonal regeneration but restricts the spread of brain damage (Barreto et al., 2011; Kang and Hébert, 2011; Xiong et al., 2011; Adelson et al., 2012). In this aspect, reactive gliosis has been associated with several neuropathologies such as AD, Parkinson (PD), Multiple Sclerosis, Batten disease (BD), ALS stroke, neuroinflammation, epilepsy, neurotrauma, brain hemorrhage, perinatal asphyxia, CNS tumors, retinal ischemia and diabetic retinopathy (Barreto et al., 2011; Phatnani and Maniatis, 2015; Rama Rao and Kielian, 2015; Pekny and Pekna, 2016). Unfortunately, to our knowledge, there are no computational models that address this important process, making it an interesting field for future development.

### Astrocytic Metabolism and Neuronal Coupling

Astrocytes are of crucial importance for brain metabolism, due to their intimate interactions with both neurons and endothelial cells, therefore it is not surprising that they have complex biochemistry that includes the transport of several biochemical metabolites through specific transporters, and metabolic shuttles (Souza et al., 2019). For example, astrocytes can store glycogen in cytoplasm granules and supply neurons, both during normal and pathological conditions with energetic molecules such as lactate, produced through the glycolytic pathway (Barreto et al., 2011; Souza et al., 2019). Moreover, astrocytes are important in *de novo* synthesis of GABA and glutamate, as they express pyruvate carboxylase and high-affinity excitatory amino acid transporters (EAATs) such as GLAST and GLT-1. The recycling of GABA and glutamate in turn is used by astrocytes to produce great amounts of the antioxidative enzyme glutathione from glutamate and cysteine. This enzyme is used in the conversion of the methylglyoxal into d-lactate by glyoxalase and is also exported to neurons to increasing their antioxidant defenses (Giordano et al., 2009; Barreto et al., 2011; McBean, 2017). In this aspect, astrocytes produce additional antioxidant molecules such as superoxide dismutase (SODs) and ascorbate which are important in the decrease of reactive oxygen species (ROS) following brain damage (Barreto et al., 2011; Zaghloul et al., 2014).

**Figure 1:**
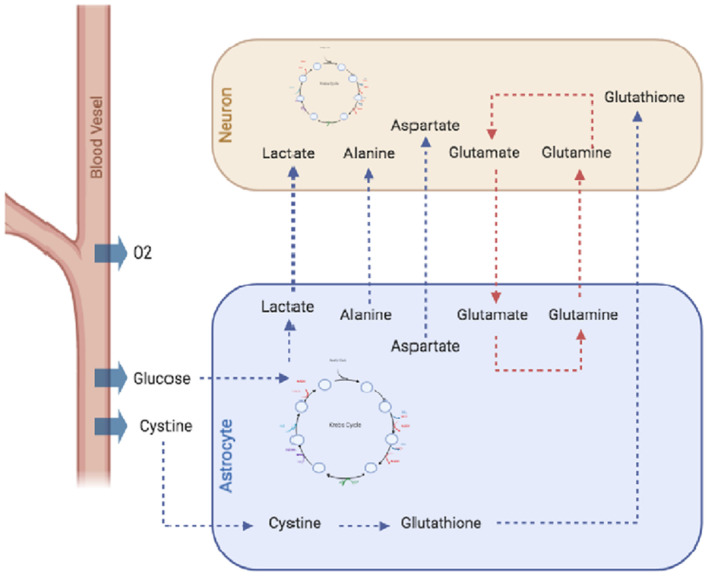
Metabolic interactions between astrocytes and neurons. Astrocytes, supply neurons with several metabolites, including amino acids like alanine and aspartate, antioxidant molecules like glutathione or lactate which is metabolized by astrocytes from glucose through the glycolytic pathway and exported to the extracellular space and internalized by neurons. Moreover, astrocytes are important in *de novo* synthesis of neurotransmitters GABA and glutamate, as they express pyruvate carboxylase, and high-affinity excitatory amino acid transporters (EAATs). Finally, astrocytes also recycle the excess of glutamate from the extracellular space, thus preventing neuronal excitotoxicity.

Finally, it is important to mention the astrocytic release of gliotransmitters like ATP, D-serine, and glutamate, which are fundamental in the regulation of synaptic function and development, paracrine activity, and suppression of synaptic transmission (Harada et al., 2016). Gliotransmitter release is modulated through changes in the intracellular calcium concentration ([Ca^2+^]_i_) and the subsequent release of synaptic-like vesicles that involve a tightly controlled regulation mechanism (Harada et al., 2016). Many of these astrocytic features have been studied using computational models of different kinds (e.g., single-cell models, metabolic reconstructions, dynamic models) due to their importance for neuronal physiology and pathology (Figure 1).

## Computational Models of Astrocytes

**Figure 2:**
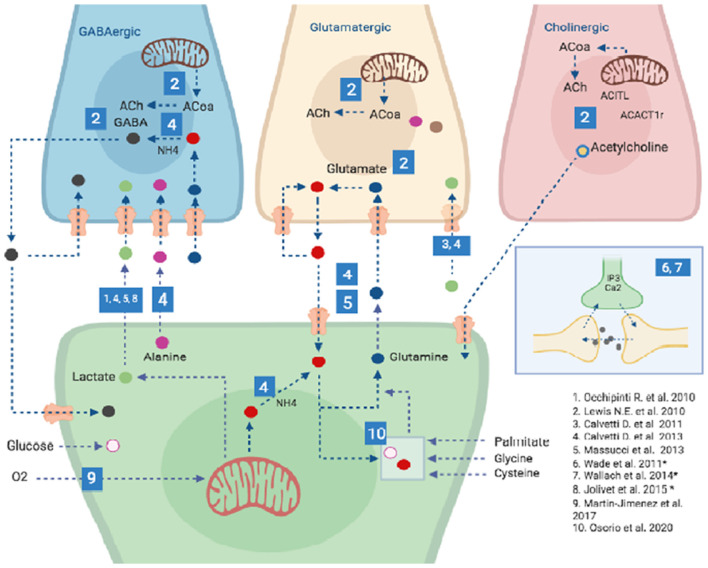
Computational models of astrocytes. Different types of astrocytic computational models have been developed recently including Single-cell astrocytic models, such as those created by 9 and 10; Specific neuron type-astrocyte coupled models (specifically GABA, Glutamate and Acetylcholine producing neurons), such as those created by 1–6 and General neuron-astrocyte coupled models such as those developed by 6-8 (and marked with an *), two of these model (6 and 7) have approved the synapses in tripartite model approach. For details on each of these models please refer to Supplementary Table S1. Other models are detailed in the text. It is important to highlight that the great majority of models developed so far have been focused on the Lactate shuttle between Astrocytes and GABAergic neurons and only one model has accounted for Choliner gic nerve cells energy metabolism and its interplay with Astrocytes. Blue numbered squares refer to a specific publication (lower right corner of the image).

In silico brain, models can integrate experimental data with computational approaches, providing a powerful framework for the understanding of brain functions. Moreover, they can be used successfully for testing hypotheses and to guide wet-bench research, thus generating novel insights on brain metabolism under normal and pathological conditions (Lewis et al., 2010; Hyduke et al., 2013). Due to the critical importance of astrocytes for brain homeostasis, neuronal protection, and metabolic regulation, there has been a growing interest in the study of astrocytic functions and metabolism through the use of computational tools, models and databases (Li et al., 2010; Volman et al., 2012; Linne and Jalonen, 2014; Sertbaş et al., 2014; Oschmann et al., 2016; Martín-Jiménez et al., 2017; Manninen et al., 2018). Numerous types of astrocytic computational models (Figure 2) with growing levels of complexity (e.g., number of reactions and metabolites, and the number of compartments) have been developed in the last years, manly based on the models by De Young and Keizer (1992), Li and Rinzel (1994) and Höfer et al. (2002). These models allow some model’s developments to study the role of astrocytes. These include single-cell astrocytic models, dynamical models (most often in calcium dynamics, synchronization, information, and plasticity, among others), astrocyte network models, neuron-astrocyte interaction networks, complete-scale metabolic reconstructions, among others (Volman et al., 2012; Linne and Jalonen, 2014; Sertbaş et al., 2014; Oschmann et al., 2016; Martín-Jiménez et al., 2017; Manninen et al., 2018). However irreproducible data is a considerable problem among the developers for the astrocyte models (Manninen et al., 2018).

Finally, it is important to highlight some significant features related to the construction of computational metabolic models. One of the most important developments in metabolic models is the construction of a mathematical model, that can represent interactions between the different components in a biological system (ions, molecules, macromolecules, etc.). Most of these models are based on ordinary differential equations (ODEs), which reflect the effects of variation of the biological processes in time, such as the behavior of enzymes, or the activation of signaling pathways (Ji et al., 2017). However, in some cases, does not exist reliable information for the kinetic parameters in a set of reactions, due to experimental difficulties (Gratie et al., 2013). In those cases, sometimes it is possible to apply approaches based on the steady-state of the model, such as flux balance analysis (FBA, Gratie et al., 2013). In the following sections, we explore some important categories of computational models used in astrocyte research, including dynamical models, astrocyte-neuron interactions, Genome-scale reconstructions, and computational models of glioblastoma.

### Dynamical Models of Astrocytes

Most of the biological phenomena are dynamical processes both in space and time; therefore, some computational models known to simulate them have taken into account those dynamical components whenever it is possible (Knüpfer and Beckstein, 2013; Nielsen, 2017). In this aspect, dynamical computational models can describe changes of a particular process (e.g change in a metabolite concentration) over time, providing detailed solutions for the equilibrium and transient states of the process from any initial condition (Kim et al., 2018). Moreover, a dynamic modeling approach is a useful tool for establishing differences among the normal physiological states of a biological network and perturbed (e.g., pathological) ones. Some of these models have been used in the study of small metabolic networks in brain pathologies such as PD and AD (Smith et al., 2004; Tiveci et al., 2005; Poliquin et al., 2013).

As regards to astrocytes, dynamical models are mainly focused on the study of calcium-related processes, such as the release of gliotransmitters, Ca^2+^ wave propagation, modulation of synaptic transmission, neurovascular coupling, long-term potentiation/depression, among others (Lecca and Lecca, 2008; Wade et al., 2011; Chander and Chakravarthy, 2012; Lecca and Lecca, 2013; Taheri et al., 2017). For example, Lecca and Lecca (2008) developed a simplified model (part of generic models where they did not specify model astrocytes in the brain) of the intercellular regulation of glucose metabolism through Na^+^ and Ca^2+^ waves, and their rate in time in astrocytes. This initial model used only nine ODE and depicted how the influence of Ca^2+^ waves on Na^+^ waves affected the rate of glucose consumption. This model was further expanded with 10 additional equations that quantified the oscillations of Na^+^ and Ca^2+^ on the rate of glucose consumption (Lecca and Lecca, 2013). Moreover, the upgraded model used experimental results, from Positron Emission Tomography (PET) images, from 31 patients to calibrate the kinetic rates constants of the simulation parameters. Similarly, Komin et al. (2015) and Taheri et al. (2017) developed a mathematical model of the IP3-dependent Ca^2+^ response in astrocytes, a mechanism that is involved in the process such as gliotransmission, modulation of transporters and genetic expression. However, gliotransmission and its underlying mechanisms still have much controversy (Taheri et al., 2017). Taheri et al.’s (2017) model also integrated quantitative data of Ca^2+^ imaging from mice brain slices, stimulated with ATP for 63 ms. Importantly, Komin et al. (2015) and Taheri et al. (2017) presented a reaction-diffusion model and a reaction model on the somatosensory cortex, respectively. Both computational models examined factors as a different receptor or Sarco/ER Ca^2+^ mechanisms that increase in Ca^2+^ concentrations in the cell, Ca^2+^ dependent signaling pathways that govern the production and release mediators from astrocytes across the temporal dynamics of IP3, the relative flux rates through Ca^2+^ channels and pumps, and the calcium distribution between astrocyte somas and processes (Taheri et al., 2017). Komin et al. (2015) and Taheri et al. (2017) showed that their models were different and the results of the reaction-diffusion model behave similarly to the experimental data, but their reaction model showed the opposite. Also, these authors found out that the channels and the pumps were necessary for log-lasting responses, as well as for stable oscillations with different parameter values of the channels and the pumps. In summary, these results show the importance of dynamical models in the understanding of metabolic processes in a time dimension and its influence on brain Modulation. Several perspectives, such as stochastic methods, have been developed for reaction and reaction-diffusion models, but most realistic simulations are provided by discrete-state stochastic reaction-diffusion methods, as well as the minute details of temporal and spatial scales, location of astrocytes and the developmental stage of an animal.

Since many studies have discussed extensively the dynamic models of calcium and their characteristics, we will not discuss it further in the present review (Volman et al., 2012; Linne and Jalonen, 2014; Oschmann et al., 2016; Manninen et al., 2018).

### Computational Models of Astrocyte-Neuron Interactions

As above mentioned, it is important to emphasize the pivotal importance of the interactions between neurons and astrocytes, which are fundamental for synaptic activity, brain homeostasis, energy metabolism, volume regulation and neuroprotection (Hamby and Sofroniew, 2010; Cabezas et al., 2014; Posada-Duque et al., 2014; Oschmann et al., 2016). In this aspect, a great number of models focusing on the metabolic interactions between neurons and astrocytes have been developed, with increasing levels of complexity and biochemical pathways involved (Gruetter et al., 2001; Gruetter, 2002; Lewis et al., 2010; Occhipinti et al., 2010; Massucci et al., 2013; Sertbaş et al., 2014; Oschmann et al., 2016). These models can be categorized in two groups; the first group is related with the study of neurotransmitter transport and exchange (Gruetter et al., 2001; Gruetter, 2002; Chatziioannou et al., 2003), and the second group focused in the analysis of the metabolic changes during brain damage and neurodegeneration (Lewis et al., 2010; Occhipinti et al., 2010; Sertbaş et al., 2014). In this review article, we focus on the second group of models, due to their relevance in the study of metabolic networks from a systemic approach.

An earlier model by Chatziioannou et al. (2003) employed flux analysis to study the glutamate release between astrocytes and neurons. This small model integrated 16 metabolic reactions with experimental results from rat or mouse brain preparations, showing the importance of phosphate activated glutaminase and aspartate aminotransferase in glutamate metabolism (Chatziioannou et al., 2003). A bigger model by Occhipinti et al. (2008) used 75 reactions in five subcellular compartments to assess the astrocyte-neuron lactate shuttle (ANLS), which is essential for the energetic supply of neurons. In this aspect, their model proposes that ANLS is used by glutamatergic neurons to bypass glycolysis impairment during energetic demanding processes (Occhipinti et al., 2008). A subsequent model by the same group (Occhipinti et al., 2010) studied the astrocytic interactions with GABAergic neurons through Bayesian statistics and Flux Balance Analysis. One interesting result of these experiments was that, regardless of neuronal activity state, the reaction fluxes associated to the glutamine flux from astrocytes to neurons, as well as the glucose uptake and the glycolytic activity, did not present a significant variation due to an observed depletion of glutamate (Glu), Aspartate (Asp) and glutamine (Gln) in astrocytes and an accumulation of these metabolites in the neuron. Additional models from this group (Calvetti and Somersalo, 2011, 2013; MacGillivray et al., 2017) explored the glutamate/GABA-glutamine cycling in astrocytes, GABAergic and Glutamatergic neurons. These general studies used steady-state models and were focused on specific aspects, such as reciprocal astrocyte-neuron interactions and signaling processes such as glutamate. Moreover, in these cases, tracing metabolites, ion channels, pH, ionic currents, or any other variables were not modified, due to the size of omic datasets and the difficult to model metabolic interactions using existing approaches. The difficulty of performing experimental studies of metabolic changes in brain cells plays an important role to decipher the contribution of its variables in disease or health. In the long-term, these combined studies provided potential pharmacological targets in pathologies associated with alterations in neurotransmitter release such as epileptic seizures, hepatic encephalopathy, dementia, among others (Calvetti and Somersalo, 2013). But, half of the models were so-called generic models because they not describe any specific anatomical location (Manninen et al., 2019). In this perspective, these authors found out that the present models could be missing details, moreover, the experimental evidence suggests that reciprocal signaling, functional state, interaction between different cell types, among other data, could propose the most realistic simulations.

Indeed, Lewis et al. (2010) assessed the metabolic interactions between astrocytes and three different neuronal types (glutamatergic, GABAergic, and cholinergic) in the context of AD. To our knowledge, this was the first robust GSMM of neurons interacting with astrocytes and integrated over 1,000 reactions for each type of neuron, 987 metabolites, and 403 genes. Their results showed that in AD development, there was a significative decrease in glycolysis, tricarboxylic acid cycle (TCA), oxidative phosphorylation and the expression of the malate-aspartate shuttle in some regions of the brain such as the posterior cingulate cortex (PC), the middle temporal gyrus (MTG) the hippocampus and entorhinal cortex (Lewis et al., 2010). Moreover, Sertbaş et al. (2014) developed an analogous astrocyte-neuron model to identify potential biomarkers from transcriptomic data for six neurodegenerative diseases (AD, PD, ALS, MS, HD, and schizophrenia). The model comprised 630 reactions (571 internal, 59 exchange; 253 for neurons; 299 for astrocyte and 59 exchange of metabolites), 570 genes and 524 metabolites (Sertbaş et al., 2014). They identified significant changes in the TCA cycle, lipid and amino acid metabolism, and changes in oxidative stress associated with the studied diseases. Moreover, they also identified some transcription factors, like KLF4, USF1, and SP1 that were overexpressed in those pathologies.

Using a different approach, DiNuzzo et al. (2017) generated a metabolic network of the energetic metabolism interactions between astrocytes and neurons, focused on the stoichiometric relationship linking glutamatergic neurotransmission to Na^+^ and K^+^ ionic currents. Importantly, the results were consistent with 13 C-MRS experimental measurements for cerebral glucose, oxygen, pyruvate dehydrogenase, and the rate of astrocytic pyruvate carboxylase from rat brains. These combined results show the importance of astrocyte-neuronal models for a greater understanding of the energetic metabolism of the brain and the associated alterations that take place in pathological conditions (Supplementary Table S1). In summary, the models discussed above represent astrocyte-neurons interactions but did not describe their location in the brain (Manninen et al., 2019). Moreover, the compartmentalization information of the processes between, within, and outside cells with transcriptomic, proteomic, and metabolomics datasets can allows that the models with metabolic coupling and synergistic activities predict more accurately true cellular functions.

### Genome-Scale Astrocytic Models

A metabolic reconstruction is a mathematical model that represents the entire network of metabolic reactions from a specific organism (Thiele et al., 2013). Several metabolic reconstructions have been done in different organisms including bacteria, archeas, yeasts, plants, animals, and humans (Duarte et al., 2007; Oberhardt et al., 2009; Thiele et al., 2013). These reconstructions are built from several sources, including the integration of biological databases, high-throughput omic data, and literature-based evidence (Najafi et al., 2014). Moreover, these reconstructions have been used to explore the metabolic features of different cells both in health and disease and predict their behavior under pathological conditions or specific stimuli (Najafi et al., 2014; Martín-Jiménez et al., 2017). Both Recon 1 and Recon 2 are manually-curated genome-scale models of human metabolism with several sub-cellular compartments (mitochondria, Golgi, etc), thousands of reactions and associated genes (Duarte et al., 2007; Thiele et al., 2013). Subsequent models of 65 cell-types from different tissues were generated by using expression data from Human Protein Atlas and Recon 2, including kidney, liver, heart, pancreas, brain and dopaminergic neurons (Uhlen et al., 2010; Büchel et al., 2013; Thiele et al., 2013).

Furthermore, in the last years, researches made brain-specific genome-scale metabolic networks that can help to interpret results across computational approaches, which with the use of multi-omics data allows the discovery of molecular pathways that are affected due to a disease state. Briefly, multi-omics approaches make use of diverse layers of biological information to better inform a metabolic model. These layers of information are typically composed of data obtained from genomics, transcriptomics, proteomics, and/or metabolomics assays. The main aim is to correlate each of this layers from the bottom (for instance genome information) up to upper layers (for instance metabolomics or proteomics data) so the presence of a gene in the genome can be traced through its expression in transcriptomics data to its functional state in proteomics data. Nevertheless, an effective correlation of several layers of biological information is challenging, mainly because of the time frame in which each of them takes place in the cell, moreover when a steady state is assumed. A typical approach is to integrate one or two of these layers. Martín-Jiménez et al. (2017) and Osorio et al. (2020) developed a genome-scale reconstruction of human astrocytes based on transcriptomic data, obtained from human fetal cortical astrocytes, and the human metabolic Atlas (HMA, Pornputtapong et al., 2015) to study the astrocytic behavior during normal conditions and brain under injury conditions (Supplementary Table S1 and Figure 2). Martín-Jiménez et al. (2017) reconstructed the astrocyte-specific model using omics data to simulate normal (healthy) and ischemic conditions by decreasing oxygen consumption. This model contains 5,007 metabolites and 5,659 reactions, which are found in eight cellular compartments (extracellular, cytoplasm, mitochondria, endoplasmic reticulum, Golgi apparatus, lysosome, peroxisome, and nucleus). The model also includes several metabolic reactions of amino acids, lipids, carbohydrates, purines, vitamins and cofactors, energy metabolism, and the glutamate-glutamine cycle. To simulate the ischemic conditions, a reduction of 20% of glucose intake and oxygen flux was included, resulting in important alterations on ATP production, glutamate uptake, lactate release, and others (Martín-Jiménez et al., 2017). Finally, the authors identified significant changes in some antioxidant enzymes, such as catalase, superoxide dismutase, and glutathione peroxidase, associated with the prevention of brain injury during ischemia (Martín-Jiménez et al., 2017).

Importantly, these specific-models as well as the other models can be used as scaffolds for analyses to computational hypotheses, because they integrate multi-omic data from neurodegenerative diseases. For example, Osorio et al. (2020) developed an astrocyte-specific model to test the effects of tibolone during metabolic inflammation by palmitic acid-induced toxicity and the effects of tibolone treatment. The authors performed a model composed of 1,262 unique genes, 1,950 metabolites, and 2,747 biochemical reactions. Metabolic simulations were made from three different metabolic scenarios (“healthy” scenario, an induced inflammation by palmitate scenario, and tibolone treatment under inflammation scenario). These authors found out that the present model and the results are similar to those previously reported from astrocyte models and the previous experimental studies (Sertbaş et al., 2014; Martín-Jiménez et al., 2017). Moreover, this model allows us to identify the metabolic changes between the three metabolic scenarios and its responses to tibolone, as well as, protective effects against inflammation, oxidative stress, and metabolic dysregulation. This genome-scale model of the astrocyte can be further explored in the study of metabolic alterations during other brain pathologies. Moreover, these models and their integration with multi-omics technologies are useful in the development of medical therapies.

## Computational Models for Other Glial Cell Types

Other cell types such as microglia, oligodendrocytes, ependymal cells, Schwann cells, and Mueller cells have also been characterized as part of the neuroglia (Souza et al., 2019). Although these cells are highly important for neuronal support, immune modulation, and production of the cerebrospinal fluid (CSF), only a few computational models have been done concerning their functions and regulation (Anderson et al., 2015).

Microglia cells are specialized macrophages of the CNS and have a major contribution in neurogenesis, immune response against infectious agents, and neuroinflammation (Lannes et al., 2017). Regarding the importance of microglia on neuroinflammation, Anderson et al. (2015) developed a cytokine signaling network for the regulation of TNFα, IL-6, IL-10, TGFβ, and CCL5 in response to bacterial lipopolysaccharides (LPS). They found that both TGFβ and IL-10 exert divergent effects on adaptation for TNFα and that the inhibition of IL-10 reduced early negative feedback that results in enhanced TNFα-mediated TGFβ expression (Anderson et al., 2015). This model was further expanded with the use of *in vivo* cytokine profiles from mice injected with LPS from *E. coli* integrated with the computational cytokine signaling network (Anderson et al., 2017). Their results showed an important association between the genetic expression of cytokines and the microglial morphology that could be used in the study of brain injuries and neuroinflammation.

Oligodendrocytes are myelinating cells of the CNS, and provide a supporting role for neurons, as well as trophic support through the release of GDNF, IGF-1, and BDNF (Philips and Rothstein, 2017). Different models of oligodendrocyte myelination and their electrical properties have been developed in the last years (Richardson et al., 2000; Bakiri et al., 2011; Walsh et al., 2017). For example, Bakiri et al. (2011) studied the action potential propagation of myelinated axons in the *corpus callosum* and the cerebellar white matter, finding important differences in the myelinating axons, the conductance of the intermodal membranes and the length of the internode spaces that can affect the speed of action potential propagation. Similarly, Walsh et al. (2017) studied the optimal distribution of myelin, regarding the sodium ion channel density in the membrane, using a simplified model of a single axon. Their results showed the importance of both myelinated and non-myelinated segments in the axon, for an appropriate conduction velocity of the action potential, thus assessing the pivotal importance of oligodendrocytes for this process.

Ependymal cells, also known as ependymocytes, are involved in the production of CSF, which protects the brain from mechanical injuries, regulate metabolite concentration and acts as a water reservoir (Kurtcuoglu, 2011; Buishas et al., 2014). In this aspect, Buishas et al. (2014) developed a computational model for the production of CSF based on experimental evidence of osmotic pressure gradients of different brain compartments, with potential applications in Hydrocephalus and brain edema (Buishas et al., 2014). Using a different approach, Meste et al. (2015) used an image-based computational simulation of ependymal ciliary beating frequency (CBF) on the modulation of CSF flux and metabolic composition, which can affect brain homeostasis (Meste et al., 2015). These combined results show the paramount importance of other glial cells in the study of brain physiology and their applications in health science and pharmaceutical development (Table 1).

**Table 1:** Selected glial cell computational models, in microglia, oligodendrocytes, and ependymal cells.

Reference	Method	Cell type	Conclusions
Anderson et al. (2015)	Dynamical	Microglia	TGFβ and IL-10 exert divergent effects on adaptation for TNFα.
Anderson et al. (2017)	Dynamical/integrative	Microglia	Changes in morphology are associated with cytokine network dynamics.
Richardson et al. (2000)	Computer-based cable models	Oligodendrocyte	Three models for myelin influence on axonal excitation and insulation.
Bakiri et al. (2011)	Computer-based cable models Rat brain slices	Oligodendrocyte	Differences in the myelinating axons, the conductance of the intermodal membranes, and the length of the internode spaces can affect the speed of action potential.
Walsh et al. (2017)	Simplified axonal model	Oligodendrocyte	Sodium-ion channel number/density has a strong influence on the optimal pattern of myelin and the conduction velocity.
Buishas et al. (2014)	Literature-based, dynamical	Ependymal/CSF	A computational model for the production of CSF based on experimental evidence of osmotic pressure gradients
Meste et al. (2015)	Computational simulation of CSF based on video-microscopy images from mice brain.	Ependymal	Computational simulation of ependymal ciliary beating frequency (CBF), on the modulation of CSF flux and metabolic composition.

However, the large heterogeneity within individual cell types as oligodendrocytes, microglia cells, among others, makes more complicated the development and description of models by specific neuron-glial interactions. These cells are present in all regions of the brain in large numbers, but they are not uniformly distributed, so only a few computational models performed the record of them (De Pittá, 2020). In oligodendrocytes, it has been demonstrated that these cells and their axons should be considered as functional units. But overall, the low information of the account of fine-tuning of AP (action potential) dynamics, synaptic processing, axonal conduction, two-way dynamic signaling from neurons to glia and vice versa, age of myelination, the brain compartmentalization among others, may suggest dynamical states that are not taken into account in models and these are important in neuronal interactions and could be used to simulate different computational purposes, within the time and spatial scales that include potentially relevant effects in the structure and function (physiology) in the healthy or injury brain.

## Multi-omic Approaches in Astrocytes and the Gut Microbiome

With the advent of high-throughput technologies, it is possible to study brain metabolism from a systemic point of view that includes thousands of biochemical reactions with complex metabolic networks (Allaman et al., 2011; Bélanger et al., 2011). For instance, pathway analysis methodologies have been valuable tools for analyzing changes in groups of genes and proteins on different biological scenarios. These approximations are based on previous knowledge of functional relationships to understand cells or organisms as a whole at different levels of complexity (Antony et al., 2012; Altaf-Ul-Amin et al., 2014). The multi-omic approach and its application in single-cell data modeling have been used to investigate the molecular interactions. Moreover, a system-level represents one of the key tools to examine the transcriptional, post-translational, and epigenetic profiles (Blencowe et al., 2019).

Regarding astrocytes, some multi-omic approaches have been done in the context of diseases such as Alzheimer neuroinflammation and glioblastoma (Currais et al., 2015; van Gijsel-Bonnello et al., 2018; Rosenberg et al., 2017; González-Ruiz et al., 2019; Rocchio et al., 2019). For example, Rocchio et al. (2019) combined shotgun proteomics, genetic expression, calcium signaling, and a computational protein network from immortalized hippocampal astrocytes to establish a highly refined model of AD. Additionally, Rosenberg et al. (2017) employed several molecular methodologies (Single nucleotide polymorphism array, expression arrays, exome sequencing, and RNA sequencing) to study the molecular landscapes from 10 human GBM tumors and a glioblastome derived cell line. They found 1,988 somatic mutations, the inhibition of immune pathways, and changes in several cell cycle and DNA repair pathways (Rosenberg et al., 2017; Miranda et al., 2018). Indeed, Currais et al. (2015) and Marttinen et al. (2019) integrated a multi-omics approach to investigate the interaction network assembly for associated gene products between biological processes and the role of post-translational modifications, phosphopeptide expression patterns associated with cell types. Both computational analyses showed that there is a decrease in gene product expression patterns, activation, and an increase in transcription genes, an abundance of protein, among others. These processes are involved in biological processes related to inflammation, platelet aggregation, and cell-matrix adhesion that could indicate the activity of microglia and astrocytic cells in the up-down-up expression profiles for proteins associated to inflammation, activation of the innate immune system in age, compartmentalization, and AD in the brain. These studies show how the integration of different omics technologies provides the means to characterize biological systems. Due to the results with high heterogeneity in some cell-types and regions of the brain, the authors propose the cell-type and associations enriched analysis and the enrichment of transcriptomic and proteomic datasets. This long-term information could be a powerful approach, considering that many authors coincide with most of the models developed, that have been derived from bulk tissues. In this sense, these bulk tissues generate generic models that represent the average activities of all cell populations, and cannot capture the unique behavior and interactions of individual cells in both space and time-specific processes.

Finally, the importance of gut microbiome has been recently addressed in neuroscience through the influence exerted by the gut-brain axis on neuroinflammation and neurodegeneration (Janakiraman and Krishnamoorthy, 2018; Ma et al., 2019). In this aspect, different studies have shown that metabolites from the gut flora can generate alterations and can contribute to various inflammatory diseases, *via* regulation and increase of metabolites that increase the activation of astrocytes through the modulation of toll-like receptor 4 (TLR4) and aryl hydrocarbon receptors (Boillot et al., 2015; Janakiraman and Krishnamoorthy, 2018; Ma et al., 2019). Activation of astrocytes by metabolites generated by bacterial species as Akkermansia and Parabacteroides increased the release of TGFα and VEGF-b, which modulate the astrocytic response to inflammation injure (Wang et al., 2018; Ma et al., 2019). Importantly, these findings and other studies related to the microbiome are a start point for the development of models of the interaction between cell-types and microbiome. In this case, some computational models of the gut microbiome have been developed (Sung et al., 2017; Dovrolis et al., 2019). For example, Sung et al. (2017) developed a system-level framework composed of 570 microbial species and three types of gastric cells. However, further studies are needed to establish a computational network of the metabolic interactions between astrocytes and the gut microbiome, where these models capture behavior, diets, specie-enrich, metabolic product, and interaction with cell-types (as microglia and astrocytes), among others. Furthermore, modeling and the application with omic data are a direction for future studies.

## Conclusions

Taking into account the growing evidence that shows how astrocytic functions are pivotal for brain metabolism and homeostasis, it is not surprising to find a major increase in computational models and methodologies dealing with the study of astrocytes (Oschmann et al., 2016; Manninen et al., 2018; Sertbas and Ulgen, 2018; Souza et al., 2019). Although most of the existing computational models have been focused in calcium dynamics and calcium-related functions, such as gliotransmitter release and propagation of calcium waves (Oschmann et al., 2016; Manninen et al., 2018), there are many astrocytic metabolic reconstructions based on FBA and related methods that have assessed the complex metabolic networks in astrocytes or astrocyte-neuron interactions (Cakir et al., 2007; Lewis et al., 2010; Occhipinti et al., 2010; Sertbaş et al., 2014). Importantly, there is only one complete genome-scale reconstruction of the human astrocyte based on genomic, transcriptomic, and biochemical data, which compares normal astrocytic metabolism with ischemic cells (Martín-Jiménez et al., 2017). It is important to also highlight the need of extending our knowledge on the interactions of astrocyte with other cell types, such as Cholinergic and Dopaminergic neuron cells since more efforts have been directed towards its interaction with GABAergic and Glutamatergic neuron types and a more comprehensive view could provide better insights into astrocyte functioning.

The availability of multi-omics data and it is seamless integration in genome-scale computational models is also a direction that, although is already applied, still have room for improvement and research. In that direction, our laboratory has been directing efforts on the development of better techniques for such integration that could be freely accessed through a unified web platform.

In summary, computational models and methods provide valuable insight into the underlying mechanisms of astrocytic functions, bringing us closer to an integrated understanding of biological processes and related pathologies through the analysis and interpretation of experimental results. More robust models are needed to assess a complete understanding of astrocytic importance for the brain and its influence on health and disease.

## Author Contributions

JG and AP conceived the presented idea. AP, AA, GB, and CM-J developed the theory and performed the computations. AA and AA-R verified the analytical methods. JG and AP encouraged AA, GB, CM-J, and AA-R to investigate conceptual and critical advances in astrocyte computational models and supervised the findings of this work. All authors contributed to the article and approved the submitted version.

## Conflict of Interest

The authors declare that the research was conducted in the absence of any commercial or financial relationships that could be construed as a potential conflict of interest.
