# Supplementary material for: Advances in Astrocyte Computational Models: From Metabolic Reconstructions to Multi-omic Approaches
Source: Front Neuroinform. 2020 Aug 7;14:35. doi: 10.3389/fninf.2020.00035 (PMC7426703; doi:10.3389/fninf.2020.00035)
Supplement: Supplementary file 1 [file Table_1.DOCX]

***Supplementary Material***

# Tables

## Supplementary Tables

## Supplementary Table 1. Comparison of selected literature/large-scale metabolic reconstruction models of the neuron-astrocyte cellular complexes developed from the system biology perspective.

| **References** | **Year** | **Reconstruction** | **Method** | **RXNs/** | **Metabolites** | **Type-cell** | **Model details** | **Outcomes** |
| --- | --- | --- | --- | --- | --- | --- | --- | --- |
|  |  |  |  | **Equations** | **(unique)** |  |  |  |
| [Occhipinti R, Somersalo E, Calvetti D](https://pubmed.ncbi.nlm.nih.gov/20664615/).^0^ | 2010 | Literature | Bayesian FBA | 75 | 27 | Astrocyte/GABAergic neuron | Genome scale metabolic model in Steady state with 6 compartments (Cytosol and Mitochondria in both cell types, extracellular space and blood). This model was based on Occhipinti model from 2009, which in turn was based on same author 2007 model. Authors use a FBA approach (Bayesian FBA) that approximates to the optimization problem, from the point of view of statistical inference (modeling all unknowns as random variables), which overcomes the issue of a possible false negative solution due to the choice of the solution method and not to the properties of the system. | Authors main aim was the identification and quantification of metabolic pathways describing the interaction between GABAergic neurons and astrocytes in connection with the release of GABA.  The neuronal uptake of lactate released by astrocytes was found to be coupled to neuronal activity  The redox balance is proposed as an explanation for the high oxidative phosphorylation evidenced in astrocytes. |
| [Lewis NE, Schramm G, Bordbar A, et al](https://www.nature.com/articles/nbt.1711).^2^ | 2010 | Large-scale/constraint-based (Recon1 based) | Monte Carlo sampling / FBA | 1066 | 983 | Astrocyte/Glutamatergic neuron | Genome scale metabolic model in Steady state, manual curation of data, based on literature to compartmentalize reconstruction into different cell types. Interaction between cell types is approached by transport reactions that communicate between them. Authors created three multicellular models each one representing a neuronal type (glutamatergic, GABA-ergic and cholinergic) in interaction with surroundings and astrocytes with a special focus on energy metabolism. Monte Carlo sampling was used in order to establish a feasible set of flux distributions. | Authors developed a workflow that integrates proteomics and expression data to model human metabolism. This method was applied to the study of brain energy metabolism for astrocyte and three neuron types. It was suggested that the decrease in the metabolic rate observed in some regions of the brain in Alzheimer's disease is mainly associated with the down regulation of central metabolic gene expression. |
|  |  |  |  | 1067 | 983 | Astrocyte/GABAergic neuron |  |  |
|  |  |  |  | 1070 | 987 | Astrocyte/Cholinergic neuron |  |  |
| [Calvetti D, Somersalo E.^[^](https://pubmed.ncbi.nlm.nih.gov/21176783/)^1]^ | 2011 | Literature | Bayesian FBA | 19 | 14 | Astrocyte/Glutamatergic neuron | Authors extended a basic kinetic model into a stochastic framework. The deterministic model is based in mass balance equations. Kinetic expressions include Michaelis-menten and transport rates based on Fick’s law, amongst others. Estimation of model parameters was approached by means of a Bayesian framework and Markov Chain Monte Carlo techniques. | Astrocytes produce and expel lactate, while neurons can switch from use to lactate.  The level of ATP hydrolysis in astrocytes is substantially higher than that required for the neurotransmitter cycle. |
| [Calvetti D, Somersalo E.^[^](https://www.ncbi.nlm.nih.gov/pmc/articles/PMC3792486/)^1]^ | 2013 | Literature | Bayesian FBA | 136 | 36 | Astrocyte/GABAergic and Glutamatergic neurons | Based on previous work of the same authors, this is a steady state model that uses Bayesian FBA and Monte Carlo sampling to estimate model variables. | Authors simulate major cycling between Astrocytes and Gaba and Glutamatergic neurons. Model suggests an important role of Leucine transport as an alternative to glutamine in order to replenish intracellular glutamate pool. |
| [Massucci F a, DiNuzzo M, Giove F, et al](https://www.ncbi.nlm.nih.gov/pmc/articles/PMC4021976/).^[1]^ | 2013 | Large-scale/constraint-based | Von Neumann’s model of production networks | 139 | 108 | Astrocyte/Glutamatergic neuron | Constraint based network model, with four main compartments. Authors introduce a change in FBA conditions inspired in Von Neuman’s production networks, and replaced the stationary condition (S.v = 0) with S.v > 0 for all intracellular metabolites, which allows flux vectors to generate a net production of chemical species. | Lactate transfer between the neuron and the astrocytes depends on the glucose uptake in the cell while it is independent of the glutamate / glutamine cycle, so the latter does not control the energy demand of neurons and astrocytes. |
| [Wade JJ, McDaid LJ, Harkin J, et al](https://journals.plos.org/plosone/article?id=10.1371/journal.pone.0029445)^.[1]^ | 2011 | Multiple models combined | Multiple methods | 16 | 3 | Astrocyte/Neuron | Authors describe a compartmentalized tripartite (Astrocyte and two neurons, including synapses) dynamic model which is in turn composed of other sub-models for Neuron, astrocyte, plasticity , synapse and astrocyte feedback. Each sub-model uses a different approach mainly based on ODEs. | Researchers focus on the role of astrocytes in neuronal plasticity rather than its role in nutrients delivery. This model suggest that the retrograde signaling through the astrocytes, allows an improvement in the synaptic transmission of those damaged synapses |
| [Wallach G, Lallouette J, Herzog N, et al](https://pubmed.ncbi.nlm.nih.gov/25521344/).^[1]^ | 2014 | Biophysical model (intracellular Ca+2 signaling) | Numerical methods | 6 | 3 | Astrocyte/Neuron | Authors describe a model of neuron-astrocyte interaction through synapses, coupling biophysical measures with computational simulation based on ODEs. | Astrocytes respond to a neural stimulation through transient calcium at the intracellular level. This activity in turn is associated with glutamate, since it could be inhibited through the glutamate type I metabotropic receptor agonist. |
| [Jolivet R, Coggan JS, Allaman I, et al](https://journals.plos.org/ploscompbiol/article?id=10.1371/journal.pcbi.1004036).^[1]^ | 2015 | Multiple models combined | Numerical methods for ODEs | 33 | 10 | Glutamatergic Neuron/Glia/Vasculature | The model consists of four compartments: neuron, astrocyte, capillary and extracellular space.  Astrocyte and neuron are compartmentalized into cytosol and mytochondria. In general the model is formulated through 33 differential equations. | This computational model integrates multiple time scales in which metabolic energy and exitability occur in the brain, focused on the lactate shuttle. Overall results support the idea of the ANLS (Astrocyte Neuron Lactate Shuttle). |
| [Martin-Jiménez et al.](https://pubmed.ncbi.nlm.nih.gov/28243200/) | 2017 | genome-scale reconstruction | FBA |  | 5007 | Astrocyte | Genome scale metabolic model reconstruction through the COBRA protocol. The model was assumed in Steady State. FBA based analysis. The model consist on 8 cell compartments, (extracellular, cytoplasm, mitochondria, endoplasmic reticle, Golgi apparatus, lysosome, peroxisome and nucleus). | authors performed a Genome-Scale Reconstruction of the Human Astrocyte Metabolic Network with the purpose of elucidating a significant portion of the metabolic map of the astrocyte.  Authors characterize network state between normal and Ischemic state |
| [Osorio et al](https://www.frontiersin.org/articles/10.3389/fnins.2019.01410/full). | 2020 | Genome-scale reconstruction | FBA | 1262 | 2747 | Astrocyte | Genome scale metabolic model reconstruction through the COBRA protocol. The model was assumed in Steady State. FBA based analysis. | Authors study the role of tibolone in free saturated fatty acids induced inflammation in astrocytes in three scenarios, healthy, palmitic acid induced inflammation (PAI) and tibolone + PAI. Candidate tibolone induced protective pathways were identified. |
